# Supplementary material for: Evolution of heterogeneity under constant and variable environments
Source: PLoS One. 2021 Sep 13;16(9):e0257377. doi: 10.1371/journal.pone.0257377 (PMC8437290; doi:10.1371/journal.pone.0257377)
Supplement: S1 File — Text A, Stationary solutions and their local stability in K-selection. Tex B, Derivation of the Hamilton-Jacobi-Bellman equation. (ZIP) [file pone.0257377.s001.zip › Supporting Infomation(final).pdf]

Supporting information

Text A

Stationary solutions and their local stability in K-selection

Existence of a stationary population

Let $P^{\dagger}(a,y)$ be the stationary population vector of Eq.(24). Then, using the Hamiltonian and fertility in Eqs. (25) and (26), it satisfies the following equations

$$\begin{aligned}\frac{\partial}{\partial a}P^{\dagger}(a,y) &= -H\left(a,y,u,\Gamma^{\dagger}\right)P^{\dagger}(a,y)\\ \Gamma^{\dagger} &= \left(\left\langle \gamma_{\ell'},P^{\dagger}\right\rangle\right)_{1\le\ell'\le d''}\\ P^{\dagger}(0,y) &= \nu(y)\left\langle F,P^{\dagger}\right\rangle\Big|_{\Gamma=\Gamma^{\dagger}}.\end{aligned} \tag{S.1}$$

Clearly, $P^{\dagger}(a,y)\equiv 0$ is a trivial solution. Otherwise, from the basic properties of the Fokker–Planck equation, the solution can be represented using the fundamental solution depending on the weighted stationary population size $\Gamma^{\dagger}$ as [1]

$$P^{\dagger}(a,y) = \beta^{\dagger}\int_A dx\ \nu(x)\,K^{\dagger}[v](0,x\to a,y)\,, \tag{S.2}$$

where $K^{\dagger}$ denotes the fundamental solution of

$$\left[\frac{\partial}{\partial a}+H\left(a,y,u,\Gamma^{\dagger}\right)\right]K^{\dagger}[u](s,x\to a,y)=0 \quad K^{\dagger}[u](s,x\to s,y)=\delta^{d}(x-y)\,. \tag{S.3}$$

From the renewal equation, the stationary neonatal cohort distribution is obtained by

$$\begin{aligned}P^{\dagger}(0,y) &= \beta^{\dagger}\nu(y)\\ &= \beta^{\dagger}\nu(y)\int_0^{\alpha}da\int_A d\xi\int_A dx\ \nu(x)\,F\left(a,\xi,u_a,\Gamma^{\dagger}\right)K^{\dagger}[u](0,x\to a,\xi)\\ &= \beta^{\dagger}\nu(y)\,\psi_0[u]\left(\Gamma^{\dagger}\right)\end{aligned}$$

Because of

$$\psi_0[u]\left(\Gamma^{\dagger}\right)=1,$$

and

$$\Gamma^{\dagger\ell'}=\left\langle\gamma_{\ell'},P^{\dagger}\right\rangle=\beta^{\dagger}\int_0^{\alpha}\int_A dady\ \gamma_{\ell'}(a,y,)\int_A dx\ \nu(x)\,K^{\dagger}[u](0,x\to a,y)\,,$$

$\beta^{\dagger}$ is

$$\beta^{\dagger}=\frac{\Gamma^{\dagger\ell'}}{\int_0^{\alpha}\int_A dady\ \gamma_{\ell'}(a,y,)\int_A dx\ \nu(x)\,K^{\dagger}[u](0,x\to a,y)}$$

for all $\ell'\in\{1,\cdots,d'\}$. Combining these pieces, we arrive at the stationary population as

$$P^{\dagger}(a,y)=\frac{\Gamma^{\dagger\ell'}\int_A dx\ \nu(x)\,K^{\dagger}[u](0,x\to a,y)}{\int_0^{\alpha}\int_A da'dy'\ \gamma_{\ell'}(a',y',)\int_A dx\ \nu(x)\,K^{\dagger}[u](0,x\to a',y')}. \tag{S.4}$$

Condition for local stability in stationary populations

To verify the local stability of the stationary population in Eq.(24), we consider the infinitesimal difference

$$\delta P_t(a,y):=P_t(a,y)-P^{\dagger}(a,y). \tag{S.5}$$

Substituting the equation above into Eq.(24), we obtain

$$\begin{aligned}\left[\frac{\partial}{\partial t}+\frac{\partial}{\partial a}\right]\delta P_t(a,y)&\approx -H\left(a,y,u,\Gamma^{\dagger}\right)\delta P_t(a,y)\\ &-\sum_{\ell'=1}^{d''}\delta\Gamma_t^{\ell'}q_{\ell'}\left(a,y,u,\Gamma^{\dagger}\right),\end{aligned} \tag{S.6}$$

where

$$\begin{aligned}\delta\Gamma_t^{\ell'}&:=\langle\gamma_{\ell'},\delta P_t\rangle\\ q_{\ell'}\left(a,y,u,\Gamma^{\dagger}\right)&:=\left[\frac{\partial}{\partial\Gamma^{\ell'}}H(a,y,u,\Gamma)\Big|_{\Gamma=\Gamma^{\dagger}}\right]P^{\dagger}(a,y).\end{aligned}$$

The fertility rate affected by density effects is approximated by

$$\begin{aligned}F(a,y,u,\Gamma_t)&\approx F\left(a,y,u,\Gamma^{\dagger}\right)+\sum_{\ell'=1}^{d''}\delta\Gamma_t^{\ell'}F'_{\ell'}\left(a,y,u,\Gamma^{\dagger}\right)\\ F'_{\ell'}\left(a,y,u,\Gamma^{\dagger}\right)&:=\frac{\partial}{\partial\Gamma^{\ell'}}F(a,y,u,\Gamma)\Big|_{\Gamma=\Gamma^{\dagger}}.\end{aligned}$$

From the equation above, the linearized renewal process becomes

$$\begin{cases}\delta P_t(0,y)=\delta\beta_t\nu(y)\\ \delta\beta_t=\langle F,\delta P_t\rangle\Big|_{\Gamma=\Gamma^{\dagger}}+\sum_{\ell'=1}^{d''}\delta\Gamma_t^{\ell'}\left\langle F'_{\ell'},P^{\dagger}\right\rangle\\ \delta\beta_t:=\beta_t-\beta^{\dagger}\end{cases} \tag{S.7}$$

This type of linear equation has the exponential solution

$$\delta P_t(a,y)\equiv C_{\lambda}\exp\{\lambda t\}\,\theta_{\lambda}(a,y),\quad \lambda\in\mathbb{C}.$$

Thus, by substituting into Eqs.(S.6) and (S.7), we obtain the characteristic equation discriminating the stability of the stationary population. Then, the unknown function $\theta_{\lambda}(a,y)$ satisfies

$$\left[\lambda+\frac{\partial}{\partial a}\right]\theta_{\lambda}(a,y)=-H\left(a,y,u,\Gamma^{\dagger}\right)\theta_{\lambda}(a,y)-\sum_{\ell'=1}^{d''}\langle\gamma_{\ell'},\theta_{\lambda}\rangle\,q_{\ell'}\left(a,y,u,\Gamma^{\dagger}\right), \tag{S.8}$$

and

$$\theta_{\lambda}(0,y)=\left[\langle F,\theta_{\lambda}\rangle\Big|_{\Gamma=\Gamma^{\dagger}}+\sum_{\ell'=1}^{d''}\langle\gamma_{l'},\theta_{\lambda}\rangle\left\langle F'_{\ell'},P^{\dagger}\right\rangle\right]\nu(v). \tag{S.9}$$

Because the inside of the parentheses $[\cdots]$ is constant with respect to y, the function at age zero can be separated by the constant and the initial state distribution.

$$\theta_\lambda(0,y) = \theta_\lambda(0)\,\nu(y)\,, \quad \theta_\lambda(0) \equiv \langle F, \theta_\lambda\rangle\Big|_{\Gamma=\Gamma^\dagger} + \sum_{\ell'=1}^{d''} \langle \gamma_{\ell'}, \theta_\lambda\rangle \left\langle F'_{\ell'}, P^\dagger \right\rangle.$$

Here, we introduce a new operator $U(a;s)$ that yields

$$\begin{aligned} U_\lambda(a;s)\,\phi(s,y) &:= \int_A dx\ \phi(s,x)\exp\{-\lambda(a-s)\}\,K^\dagger[v](s,x\to a,y) \quad 0\le s\le a \\ \lim_{a\downarrow s} U_\lambda(a;s) &= I, \end{aligned}$$

where $K^\dagger$ represents the same fundamental solution as that in Eq.(S.3). From the variational constant methodology, we computed the formal solution of Eq.(S.8) as

$$\theta_\lambda(a,y) = \theta_\lambda(0)\,U_\lambda(a;0)\,\nu(y) - \sum_{\ell'=1}^{d''}\langle\gamma_{\ell'},\theta_\lambda\rangle \int_0^a ds\ U_\lambda(a;s)\,q_{\ell'}\left(s,y,u,\Gamma^\dagger\right). \quad \text{(S.10)}$$

Computing $\langle\gamma_{\ell'},\theta_\lambda\rangle$ using the equation above, we obtain

$$\begin{aligned} \langle\gamma_{\ell'},\theta_\lambda\rangle &= \theta_\lambda(0)\left\langle\gamma_{\ell'}, w_\lambda^\dagger\right\rangle - \sum_{\ell'=1}^{d''}\langle\gamma_{\ell'},\mathcal{Q}_{\lambda,\ell''}\rangle\langle\gamma_{\ell''},\theta_\lambda\rangle \quad \text{(S.11)} \\ w_\lambda^\dagger(a,y) &:= \exp\{-\lambda a\}\int_A dx\ \nu(x)\,K^\dagger[u](0,x\to a,y) \\ \mathcal{Q}_{\lambda,\ell''}(a,y) &:= \int_0^a ds\ \ \exp\{-\lambda(a-s)\}\int_A dx\ q_{\ell''}\left(s,x,u,\Gamma^\dagger\right)K^\dagger[u](s,x\to a,y)\,. \end{aligned}$$

This equation is solvable, such that

$$\begin{aligned} \left(\langle\gamma_{\ell'},\theta_\lambda\rangle\right)_{1\le\ell'\le d''} &= \theta_\lambda(0)\left(\mathbf{I}+\mathbf{Q}_\lambda\right)^{-1}\mathbf{\Phi}_\lambda \\ \mathbf{I}\ &: d''\times d''\text{–Identity matrix} \\ \mathbf{Q}_\lambda &:= \left(\langle\gamma_{\ell'},\mathcal{Q}_{\lambda,\ell''}\rangle\right)_{1\le\ell',\ell''\le d''} \\ \mathbf{\Phi}_\lambda &:= \left(\left\langle\gamma_{\ell'}, w_\lambda^\dagger\right\rangle\right)_{1\le\ell'\le d''} \end{aligned}$$

Substituting this result into $\theta_\lambda(0)$ and Eq.(S.10), we have

$$\begin{aligned} \theta_\lambda(0) &= \theta_\lambda(0)\left(\psi_\lambda[u]\left(\Gamma^\dagger\right) - \mathbf{R}_\lambda^\top\left(\mathbf{I}+\mathbf{Q}_\lambda\right)^{-1}\mathbf{\Phi}_\lambda\right) \\ \mathbf{R}_\lambda &:= \left(\langle F,\mathcal{Q}_{\lambda,\ell'}\rangle\Big|_{\Gamma=\Gamma^\dagger} - \left\langle F'_{\ell'},P^\dagger\right\rangle\right)_{1\le\ell'\le d''}. \end{aligned}$$

Therefore, the characteristic roots $\lambda_k\in\mathbb{C}$ generating the exponential solution satisfy

$$1 = \psi_{\lambda_k}[u]\left(\Gamma^\dagger\right) - \mathbf{R}_{\lambda_k}^\top\left(\mathbf{I}+\mathbf{Q}_{\lambda_k}\right)^{-1}\mathbf{\Phi}_{\lambda_k}. \quad \text{(S.12)}$$

Let $\Lambda\subset\mathbb{C}$ be the set of characteristic roots given by the above characteristic equation. Then, the asymptotic behavior of the infinitesimal difference $\delta P_t(a,y)$ is given by the linear combination of the corresponding exponential solutions:

$$\delta P_t(a,y) = \sum_{k\in N(\Lambda)} C_k \exp\{\lambda_k t\}\, \theta_{\lambda_k}(a,y),$$

where $N(\Lambda)$ denotes the set of characteristic roots. If all characteristic roots $\lambda_k \in \Lambda$ have negative real parts, then the stationary population is asymptotically stable.

Text B

Derivation of the Hamilton–Jacobi–Bellman equation

Here, we verify that the HJB equation is derived from Bellman's principle.

To derive the HJB equation, it is reasonable to use a stochastic interpretation of the objective function. Subsequently, the Hamiltonian, Eq. (25) is generated by the following SDE.

$$\begin{cases} dX_a^j = g_j(a, X_a, u, \Gamma)\, da + \sum_{\ell=1}^{N} \sigma_{\ell j}(a, X_a, u, \Gamma)\, dB_a^\ell \quad (j = 1, 2, 3, \cdots, d) \\ X_0^j = y^j, \end{cases} \tag{S.13}$$

with corresponding mortality $\mu(a, X_a, u, \Gamma)$ and

$$S_{j,j'}(a,y,u,\Gamma) = \sum_{\ell=1}^{N} \sigma_{\ell j}(a,y,u,\Gamma)\, \sigma_{\ell j'}(a,y,u,\Gamma).$$

Using the solution of this SDE, Bellman's principle is formulated as

$$\begin{aligned} &\tilde{v}_r(a_0, y, \Gamma) = \\ &\sup_{u\in\mathbb{U}} \Bigg\{ \mathbb{E}_y \Bigg[\tilde{v}_r(a, X_a, \Gamma) \exp\left\{ -\int_{a_0}^{a} d\tau \ \ (\mu(\tau, X_\tau, u_\tau, \Gamma) + r) \right\} \\ &+ \tilde{\psi}_r(\Gamma) \int_{a_0}^{a} d\tau F(\tau, X_\tau, u_\tau, \Gamma) \exp\left\{ -\int_{a_0}^{\tau} d\tau' \ \ (\mu(\tau', X_{\tau'}, u_{\tau'}, \Gamma) + r) \right\} \Bigg] \Bigg\}, \end{aligned}$$

where $0 \le a_0 \le a \le \alpha$ can be deformed as

$$
\begin{aligned}
0 &= \sup_{u\in\mathbb{U}} \left\{ \mathbb{E}_y \left[\tilde{v}_r\left(a, X_a, \Gamma\right) \exp\left\{ -\int_{a_0}^{a} d\tau \;\; \left(\mu\left(\tau, X_\tau, u_\tau, \Gamma\right) + r\right) \right\} - \tilde{v}_r\left(a_0, y, \Gamma\right) \right.\right. \\
&\quad \left.\left. + \tilde{\psi}_r\left(\Gamma\right) \int_{a_0}^{a} d\tau F\left(\tau, X_\tau, u_\tau, \Gamma\right) \exp\left\{ -\int_{a_0}^{\tau} d\tau' \;\; \left(\mu\left(\tau', X_{\tau'}, u_{\tau'}, \Gamma\right) + r\right) \right\} \right] \right\} \\
&= \sup_{u\in\mathbb{U}} \left\{ \mathbb{E}_y \left[\int_{a_0}^{a} d \left(\tilde{v}_r^*\left(s, X_s, \Gamma\right) \exp\left\{ -\int_{a_0}^{s} d\tau \;\; \left(\mu\left(\tau, X_\tau, u_\tau, \Gamma\right) + r\right) \right\} \right) \right.\right. \\
&\quad \left.\left. + \tilde{\psi}_r\left(\Gamma\right) \int_{a_0}^{a} d\tau F\left(\tau, X_\tau, u_\tau, \Gamma\right) \exp\left\{ -\int_{a_0}^{\tau} d\tau' \;\; \left(\mu\left(\tau', X_{\tau'}, u_{\tau'}, \Gamma\right) + r\right) \right\} \right] \right\} \\
&\quad \left(a \to a_0 + \delta a\right) \\
&= \sup_{u\in\mathbb{U}} \left\{ \mathbb{E}_y \left[\int_{a_0}^{a_0+\delta a} ds \;\; \underbrace{\left[\frac{\partial}{\partial s} - H^*\left(s, X_s, u_s, \Gamma\right) - r \right] \tilde{v}_r\left(s, X_s, \Gamma\right)}_{\text{Feynman-Kac formula}} \right.\right. \\
&\quad \times \exp\left\{ -\int_{a_0}^{s} d\tau \;\; \left(\mu\left(\tau, X_\tau, v_\tau, \Gamma\right) + r\right) \right\} \\
&\quad \left.\left. + \tilde{\psi}_r\left(\Gamma\right) \int_{a_0}^{a_0+\delta a} d\tau F\left(\tau, X_\tau, u_\tau, \Gamma\right) \exp\left\{ -\int_{a_0}^{\tau} d\tau' \;\; \left(\mu\left(\tau', X_{\tau'}, u_{\tau'}, \Gamma\right) + r\right) \right\} \right] \right\}.
\end{aligned}
$$

From the deformation above, we consider the limit

$$
\begin{aligned}
0 &= \lim_{\delta a \downarrow 0} \frac{1}{\delta a} \sup_{u\in\mathbb{U}} \left\{ \mathbb{E}_y \left[\int_{a_0}^{a_0+\delta a} ds \;\; \left[\frac{\partial}{\partial s} - H^\dagger\left(s, X_s, u_s, \Gamma\right) - r \right] \tilde{v}_r\left(s, X_s, \Gamma\right) \right.\right. \\
&\quad \times \exp\left\{ -\int_{a_0}^{s} d\tau \;\; \left(\mu\left(\tau, X_\tau, u_\tau, \Gamma\right) + r\right) \right\} \\
&\quad \left.\left. + \tilde{\psi}_r\left(\Gamma\right) \int_{a_0}^{a_0+\delta a} d\tau F\left(\tau, X_\tau, u_\tau, \Gamma\right) \exp\left\{ -\int_{a_0}^{\tau} d\tau' \;\; \left(\mu\left(\tau', X_{\tau'}, u_{\tau'}, \Gamma\right) + r\right) \right\} \right] \right\} \\
&= \frac{\partial}{\partial a_0} \tilde{v}_r\left(a_0, y, \Gamma\right) - \inf_{u\in\mathbb{U}} \left\{ \left[H^*\left(a_0, y, u, \Gamma\right) - r\right] \tilde{v}_r\left(a_0, y, \Gamma\right) - \tilde{\psi}_r\left(\Gamma\right) F\left(a_0, y, v, \Gamma\right) \right\}.
\end{aligned}
$$

Shifting the age to $a_0 = a$, we have an equation identical to the HJB equation in the main text.

References

1. Karatzas I, Shreve SE. Brownian motion and stochastic calculus. vol. 113. Springer Verlag; 1991.
