# Supplementary material for: Evolution of heterogeneity under constant and variable environments
Source: PLoS One. 2021 Sep 13;16(9):e0257377. doi: 10.1371/journal.pone.0257377 (PMC8437290; doi:10.1371/journal.pone.0257377)
Supplement: Attachment — Submitted filename: Report.pdf [file pone.0257377.s002.pdf]

Report on the manuscript PONE-D-21-12079 ”Evolution of heterogeneity under constant and variable environmets”

The manuscript under review studies the adaptive life schedule in the absence and presence of a variable environment based on a continuous age-multi-state structured population model. The results of the manuscript sound interesting.

However, I must be honest that the topics of the manuscript is rather far from my expertise, and I am not be able to comment on the correctness of the research. Hence I will only comment on some mathematical part and presentation, but can not provide a recommendation and leave it to the editor.

Some detailed comments:

1. What is the meaning of $P_t(a, y)$ and the meaning of equation (3)? Please provide interpretation for these.
2. line 117, the citation [16] should be placed after the sentence in line 116.
3. In (4)–line 119: the integral variable y should be replaced by another one since it confuses with y on the LHS.
4. line 123, $p(t, a) := P(t, a\cdot)$: the notation $P(t, a, \cdot)$ is not consistent with the previous notation $P_t(a, \cdot)$ (the time variable t is in the subscript).
5. line 125, what is the reference ”eqrefmac2”? there should be a blank space after the bracket there.
6. line 229, ”determined in ??.”: please replace ?? by a correct reference.
7. line 266, ”(see ??).”: insert a correct reference.
8. line 157: the full stop punctuation (after (11)) should be removed.
9. Line 142, please provide a reference for the statement.
10. What is r_0 in Eq.(12)?
11. line 229, ”determined in ??.”: what is ??
12. a comma is redundant at the end of Eq (31).
13. the full stop in Eq (32) should be removed.
14. After Eq (34), ”See ??”: what is ??
15. Please explain how to obtain (41) since it is not trivial at all.
