# Supplementary material for: Evolution of heterogeneity under constant and variable environments
Source: PLoS One. 2021 Sep 13;16(9):e0257377. doi: 10.1371/journal.pone.0257377 (PMC8437290; doi:10.1371/journal.pone.0257377)
Supplement: Attachment — Submitted filename: response_to_reviewers_Plos_one3(20210808).docx [file pone.0257377.s003.docx]

**Reviewer #1:**

In this manuscript, the authors focus on the eigenfunction of an age-structured population model as fitness. The function generates an equation, called the Hamilton-Jacobi-Bellman equation, that achieves adaptive control of life history in terms of both the presence and absence of the density effect. Further, they introduce a perturbation method that applies the solution of this equation to the long-term logarithmic growth rate of a stochastic structured population model. They adopt this method to realize the adaptive control of heterogeneity for an optimal foraging problem in a variable environment as the analyzable example. The result indicates that the eigenfunction is involved in adaptive strategies under all the listed environments here. The authors reveal that fitness is closely related to the reproductive value. They show that characteristic functions play an important role in population dynamics even in constant and variable environments. Their model shows that heterogeneity is more likely to evolve in a variable environment than in a constant environment. After reading this work, I have found it interesting and technically sound. However, I still have some questions or comments on this work.

A. Thank you very much for your interest. We have endeavored to address your questions and comments both here and in the revised manuscript.

(1) In this work, the authors adopt the method to realize the adaptive control of heterogeneity for an optimal foraging problem in a variable environment as the analyzable example. In this analyzable example, I wonder what the meanings of adaptive control are and whether the adaptive control rule makes senses.

A. To clarify the meaning of adaptive control in the analyzable example, we have added a sentence stating that the control provides the optimal size growth curve maximizing the long term logarithmic growth rate (line 426 in the revised manuscript).

To show that the analyzable model is an example of our framework, we have added the description that Eq. (68) is one of the HJB equations (Eq. (35)) (line 430), and that the derivation of Eq. (68) from the reproductive value is achieved in the same way as the general HJB equation (under line 430).

(2) There are some typos in the manuscript, e.g., see line 266. I suggest the authors proof-read again and have the spelling and language checked.

A. Thank you for pointing out those oversights. We have corrected all typos and had the revised manuscript proofread by a professional English language editing service.

**Reviewer’s comments in the attachment**

1. What is the meaning of Pt(a; y) and the meaning of equation (3)? Please provide interpretation for these.
A. Thank you very much for your comments. Accordingly, we have added the following interpretations to line 115:

“Let the population vector $P_{t}\left(a, y\right)$, in which each individual follows the ingredients Eq. \eqref{sde0}, $F\left(a,y\right)$, and Eq.\eqref{dr0}, be a cohort density at age $a$ at a state $y$ in time $t$.” and “Eq. \eqref{mac1} implies that the cohort transitions dynamically for age $a$ and state $y$ at time $t$.”

2. line 117, the citation [16] should be placed after the sentence in line 116.

A. We agree with you; the corresponding change has been made.

3. In (4){line 119: the integral variable y should be replaced by another one since it confuses

with y on the LHS.

A. We agree with you; the corresponding change has been made.

4. line 123, p(t; a) := P(t; a_): the notation P(t; a; _) is not consistent with the previous notation

Pt(a; _) (the time variable t is in the subscript).

A. Thank you very much for pointing this out. The symbol has been changed in the revised manuscript.

5. line 125, what is the reference "eqrefmac2"? there should be a blank space after the bracket

there.

A. We have corrected the misrepresentation.

6. line 229, "determined in ??.": please replace ?? by a correct reference.

A. Thank you for pointing this out. We have replaced ?? with the correct reference, S.1.

7. line 266, "(see ??).": insert a correct reference.

A. Thank you for pointing this out. We have replaced ?? with the correct reference, S.2.

8. line 157: the full stop punctuation (after (11)) should be removed.

A. Thank you for pointing this out. We have removed it and moved the equation to line 147.

9. Line 142, please provide a reference for the statement.

A. Thank you for pointing this out. We have added a relevant reference (Metz and Diekmann 1986).

10. What is r0 in Eq.(12)?

A. Thank you for this question. We have added its corresponding explanation immediately after Eq. (12).

11. line 229, "determined in ??.": what is ??

A. Thank you for this question. We have replaced ?? with the correct reference, S.1.

12. a comma is redundant at the end of Eq (31).

A. Thank you for pointing this out. We have removed it.

13. the full stop in Eq (32) should be removed.

A. Thank you for pointing this out. We have replaced it with a comma.

14. After Eq (34), "See ??": what is ??

A. We have replaced ?? with the correct reference, S.2.

15. Please explain how to obtain (41) since it is not trivial at all.

A. Indeed, there was a lack of explanation for the derivation. Therefore, we explain the operation symbol after new Eq. 41 appears and the requirement formula that is the main result in [22] after Eq. 42 in the revised manuscript.

Please check whether they are satisfactory.
